# Prefrontal Cortex Oxygenation Evoked by Convergence Load Under Conflicting Stimulus-to-Accommodation and Stimulus-to-Vergence Eye-Movements Measured by NIRS

**DOI:** 10.3389/fnhum.2018.00298

**Published:** 2018-07-30

**Authors:** Hans O. Richter, M. Forsman, G. H. Elcadi, R. Brautaset, John E. Marsh, C. Zetterberg

**Affiliations:** ^1^Department of Occupational and Public Health Sciences, Faculty of Health and Occupational Studies, Centre for Musculoskeletal Research, University of Gävle, Gävle, Sweden; ^2^Institute of Environmental Medicine, Karolinska Institutet, Stockholm, Sweden; ^3^Department of Health and Caring Sciences, Faculty of Health and Occupational Studies, University of Gävle, Gävle, Sweden; ^4^School of Optometry, Karolinska Institutet, Stockholm, Sweden; ^5^Environmental Psychology, Department of Building, Energy, and Environmental Engineering, University of Gävle, Gävle, Sweden; ^6^School of Psychology, University of Central Lancashire, Preston, United Kingdom; ^7^Section of Occupational and Environmental Medicine, Department of Medical Sciences, Uppsala University, Uppsala, Sweden

**Keywords:** attention fatigue, accommodation, compensatory effort, convergence, disparity, near infrared spectroscopy (NIRS), time series analysis, visual ergonomics

## Abstract

**Purpose:** To extend our knowledge of the functional linkages between visual fatigue and regional cerebral prefrontal cortex (PFC) oxygenation, we measured time related hemodynamic changes over the right dorsolateral prefrontal cortex (dlPFC) during convergence load under conflicting stimulus-to-accommodation and stimulus-to-vergence eye movements with and without concurrent mental load.

**Methods:** Twenty healthy participants with a median age of 28 years (range: 18–44 years) fixated upon a vertical bar presented separately to the left and right eyes, using polarized filters, during four counterbalanced 10-min periods: (i) no accommodation/vergence conflict (Control, *Ctrl*); (ii) added convergence load and accommodation/vergence conflict (*Conv*); (iii) added cognitive load only (*Cog*) and; (iv) a combination of added cognitive and convergence load and accommodation/vergence conflict (*Cc*). Viewing distance was 65 cm. Non-invasive measurements of hemodynamic activity over the dlPFC were quantified by functional near-infrared spectroscopy (fNIRS). During the two-convergence load conditions, the horizontal disparity of the two bars varied dynamically from no disparity to a disparity set 20% below the individual threshold for diplopia. Cognitive load was induced by the *n*-back-2 test which required the subject to memorize and recall the changing colors of the horizontal bars and decide when a given color was the same as that occurring two colors previously. fNIRS data were averaged over 10-s windows centered at 0, 2, 4, 6, 8, and 10 min of each task, subtracted from a 20-s baseline window immediately preceding the visual task, and then represented as changes in oxygenated hemoglobin (ΔHbO_2_)_,_ deoxygenated hemoglobin (ΔHHb) and total hemoglobin (ΔtHb).

**Results:** Linear mixed model analyses showed that hemodynamic activity was systematically influenced by time (*p* < 0.001). The group-averaged time-related level of change across the viewing conditions did not differ when compared with one another (*p* > 0.05). Larger convergence eye-movement responses under conflicting stimulus-to-accommodation, and stimulus-to-vergence over time, increased ΔHbO_2_ and ΔtHb only in condition *Cc* and after 8 min of task time (*p* < 0.10 for min^-6^ and min^-8^: *p* < 0.05 for min^-10^).

**Discussion:** Collectively, our data suggest that HbO_2_, HHb, and tHb, recorded over the dlPFC with fNIRS, can be used to assay the degree to which supervisory oculomotor control processes are activated during visually deficient near work.

## Introduction

Visual fatigue, a condition of improper or reduced visual function is believed to result from the use or overuse of the eyes and has attracted and puzzled researchers from many different fields (Franco and Franco, 2001; Cai et al., 2017). A sense of eye strain, eye weakness, or eye fatigue (i.e., asthenopia, Hofstetter et al., 2000), in combination with musculoskeletal symptoms from the neck and shoulder region, has been widely acknowledged as being associated with occupational near work since the 1980s (Gobba et al., 1988; Piccoli and ICOH Scientific Committee, 2003; Blehm et al., 2005). However, none of the existing scientific, clinical, and applied research carried out thus far has focused exclusively on the specific involvement of the central nervous system, and particularly supervisory (i.e., pre-frontal) processes, in the development of visual fatigue.

Accommodative and non-strabismic binocular dysfunctions are visual disorders that affect the subject’s binocular vision and visual performance, particularly when performing work tasks requiring near vision. Due to the efforts involved in prolonged near vision, the visual system may suffer a loss of efficiency, thus hindering near visual activities and provoking visual symptoms. Binocular and accommodative anomalies may also cause the subject to avoid demanding near work and thus report no symptoms. To be able to treat and prevent the negative health consequences of visually demanding near work, potential interactions between central-cognitive load and peripheral eye-muscle load must be investigated and understood. In this context, the overall goal of the current research was to determine and describe linkages between visually deficient work conditions and their effects upon the activity of the prefrontal cortex (PFC).

The achievement of single clear vision is due to the combined use of the sensory and motor systems. The oculomotor system acts to point both eyes to the target of interest; any offset (i.e., retinal disparity) is detected visually, and is corrected by the motor system. Vergence eye movements, including both convergence and divergence, minimize retinal disparity, while accommodation of the eye-lens adjusts the focal point to the same target. Under normal viewing conditions, these accommodation/vergence responses are tightly coupled with one another. Based on the presence of the neural coupling from vergence to accommodation, accommodation increases and decreases during convergence and divergence stimulation, respectively (Schor, 1999). However, under certain circumstances, this synergy between accommodation/vergence eye-movements is disturbed. Heterophorias, such as exophoria, the divergent turning of both eyes relative to each other, as manifested in the absence of fusional stimuli, are common public health issues (Lara et al., 2001; Von Noorden and Campos Emilio, 2002; García-Muñoz et al., 2016; Hussaindeen et al., 2017). The process in which fusional reserve is used to compensate for heterophoria is known as compensating vergence. In severe cases, when heterophoria is not overcome by fusional vergence, diplopia and symptoms such as eye fatigue, pain or discomfort tend to appear (Von Noorden and Campos Emilio, 2002). In many previous studies, the symptoms of visual fatigue have been linked to “vergence efforts” associated with a compensation of a phoria during reading and near working (Iribarren et al., 2001; Marran et al., 2006; Howarth, 2011; Ovenseri-Ogbomo and Eguegu, 2016). However, the role of central supervisory processes during this type of visual effort still remain unclear.

Pivotal to the current study, which aimed to extend our knowledge of the functional linkages between visual fatigue and PFC activity, are recent advancements in the quantification of brain activity by functional Near Infrared Spectroscopy (fNIRS), which have opened new and unique avenues for investigation (Ferrari and Quaresima, 2012; Ayaz et al., 2013; Derosiere et al., 2013). Several technical advantages are offered by fNIRS compared to conventional neuroimaging methods (see e.g., Naseer and Hong, 2013, 2015). Non-invasive measurements of local cortical hemodynamics, for example, total hemoglobin (tHb), HHb, and HbO_2_, can now be performed routinely with spectrometers capable of emitting and detecting light at different wavelength intensities. It is also possible for participants to engage in experimental tasks without any movement or noise limitation constraints associated, for instance, with functional magnetic resonance imaging (fMRI). Moreover, the possibility of quantifying central hemodynamic changes to visual fatigue in subjects in an ecologically realistic sitting and gazing position is a particular advantage of fNIRS (Piper et al., 2014).

The PFC, and specifically the right dorsolateral prefrontal cortex, Brodmann area 46 (dlPFC), is a brain region of key interest in the context of vergence load and effort because of its supervisory role in oculomotor control and its involvement in visual processing (Fink et al., 1999; Barceló et al., 2000; Pierrot-Deseilligny et al., 2005; Gu and Corneil, 2014). If stimuli and/or responses are spatial, the right dlPFC is prominently activated, and the greater the mental demand required by a given task, the greater the level of activation (Fuster, 2015). Tanaka et al. (2014) suggested that the dlPFC is a key mediating structure which responds to a facilitation and inhibition system activated by physical fatigue. Information processing output is increased to compensate for the effects of mental fatigue and primary motor cortex output is increased as physical fatigue increases. The point of departure in this context is that the dlPFC should contribute to the control of fusional vergence eye-movements during visually demanding near work. Central effort, which overrides the need for rest and recuperation, to avoid deterioration in visual performance, should recruit the dlPFC (Tanaka et al., 2014). The dlPFC is also capable of allocating more mental/physical resources to carry out a given work task in the case of visually related mental fatigue (Derosiere et al., 2013; Mandrick et al., 2013).

The specific purpose of this study was to begin to identify hemodynamic changes over the right dlPFC during the additional convergence load under conflicting stimulus-to-accommodation and stimulus-to-vergence eye-movements, with and without added cognitive load (Pierrot-Deseilligny et al., 2005). This study used a specific experimental approach. The dimension of time was applied into the analyses of hemodynamic changes recorded over the dlPFC during the added convergence load. Previous studies have not considered the relationship between visual effort, fatigue and central supervisory activity and the dynamics of dlPFC activity, as quantified by fNIRS. Because many near vision tasks involve a combination of highly sustained convergence demands (Howarth, 2011) which continue for a prolonged time period, this research has several implications for translational health.

The present study explored three different interrelated research hypotheses, as detailed below:

(1) For visual near-viewing conditions, involving added convergence demands and/or added mental demands (subjecting the participants to external exposure of mental/physical stress), the effect on dLPFC should be significantly influenced by time because the added demands trigger the need for more central control (Pierrot-Deseilligny et al., 2005; Richter et al., 2015).
(2) The individual degree of vergence load (i.e., internal exposure) should impact upon PFC activity such that dlPFC oxygenation should increase more in magnitude over time among subjects that are subjected to larger levels of convergence load because these participants will allocate more central resources to execute larger amplitudes of vergence eye-movements to avoid diplopia and vice versa.
(3) The combined effects of concurrent oculomotor demand and cognitive demands on dlPFC activity should be additive. This hypothesis tests if dlPFC oxygenation is influenced by a combination of added convergence demands and added cognitive demands as compared to conditions with only added convergence demands, only added cognitive load, or no added load (Leone et al., 2017).

## Materials and Methods

Data for this study were collected as a part of a larger study examining the effect of cognitive and convergence load on the activation of the trapezius muscle, as measured concomitantly by electromyography and fNIRS. The present report includes analysis of fNIRS data related to convergence effort during conflicting stimulus-to-accommodation and stimulus-to-vergence eye-movements.

### Participants

This study featured twenty right-handed voluntary participants, who were all healthy and without any reported symptoms of central disabilities, and free from any medication related to the central nervous system (median age: 28 years; range: 18–44 years; 16 females and 4 males). To exclude participants with eye diseases, a licensed optometrist examined 15 out of the 20 participants. Based upon ophthalmic measurements, these participants were classified as having normal binocular vision and well-compensated heterophorias (see Supplementary Table S1 for visual exam). The remaining five subjects failed to comply with the instruction to schedule an optometric exam. None of the remaining 5 participants indicated any history of eye disease or history of eye-related health problems. Data from all participants were included in the statistical analysis. Informed consent was obtained from each participant. The study was approved by the Uppsala University Medical Ethical Review Board, Uppsala, Sweden (2014:366).

### Procedure and Preparations

Participants visited the laboratory on one occasion and undertook four consecutive visual tasks, each of 10-min duration. Each vision task was preceded by a baseline period (rest) when the participant sat relaxed with their eyes closed for 2-min. Statistical analyses were conducted on baseline subtracted data. The vision tasks consisted of sustained fixation on a vertical bar stimulus which was displayed on a three-dimensional (3D) screen. The distance to the screen was 0.65 m. The center of the bar was placed in the midline of the eyes, with the gaze angle approximately 15° downward. The participants fixated upon this vertical bar which was presented separately to the left and right eyes through polarized filters during the following four counterbalanced 10-min periods: (i) no accommodation/vergence conflict, in which the bars seen by the right and left eye were superimposed on each other and accommodation and convergence were both postured on the 3-D screen (Control, *Ctrl*); (ii) added convergence load (*Conv*), in which the bars seen by the right and left eye were laterally displaced to induce disparity and drive fusional vergence eye movements, which in turn induced a conflict in accommodation and vergence stimulation; (iii), added cognitive load (*Cog*) but no conflicting stimulus-to-accommodation and stimulus-to-vergence eye-movements and; (iv) a combination of added cognitive and added convergence load (*Cc*). In *Cc*, vergence eye movements were requested concurrently with a request to memorize and recall the color of the bar stimuli so that participants were simultaneously exposed to conflicting stimulus-to-accommodation, and stimulus-to-vergence eye movements, in addition to cognitive load.

### Mental Load With the *n*-Back Task

In *Cc* and *Cog* conditions, viewing consisted of 10 min of sustained mental load administered via the so-called *n*-back task (Jonides et al., 1997; Linden et al., 2003; Jaeggi et al., 2010; León-Domínguez et al., 2015). In the current study, *n*-back was implemented by requesting the subjects to memorize and recall the color of each sequentially occurring vertically presented bar and decide when a given color was the same as that occurring two colors previously. We chose *n* = 2 to emphasize a low level of “every day” cognitive load which is compatible with performing a routine intellectual activity. Each stimulus (blue, yellow, red, or green) was presented for 3.5 s with a 0.5 s inter-stimulus interval, after which a new colored stimulus appeared. A total of 170 test items were presented. If the same color reappeared, two after the one just presented (i.e., “red,” “green,” and then “red” again), participants were requested to press a low force response button. Equal emphasis was put on speed and accuracy. If the response was correct, a match was recorded. A maximum of 33 correct responses could be achieved. We monitored reaction-time (RT) in ms, the number of correct responses and the number of misses and stored these data for off-line analysis. In *Ctrl* and *Conv*, subjects were requested to ignore the changing colors of the bar stimuli, i.e., there was no memory load imposed on the participants.

### Vergence Stimulation

**FIGURE 1:**
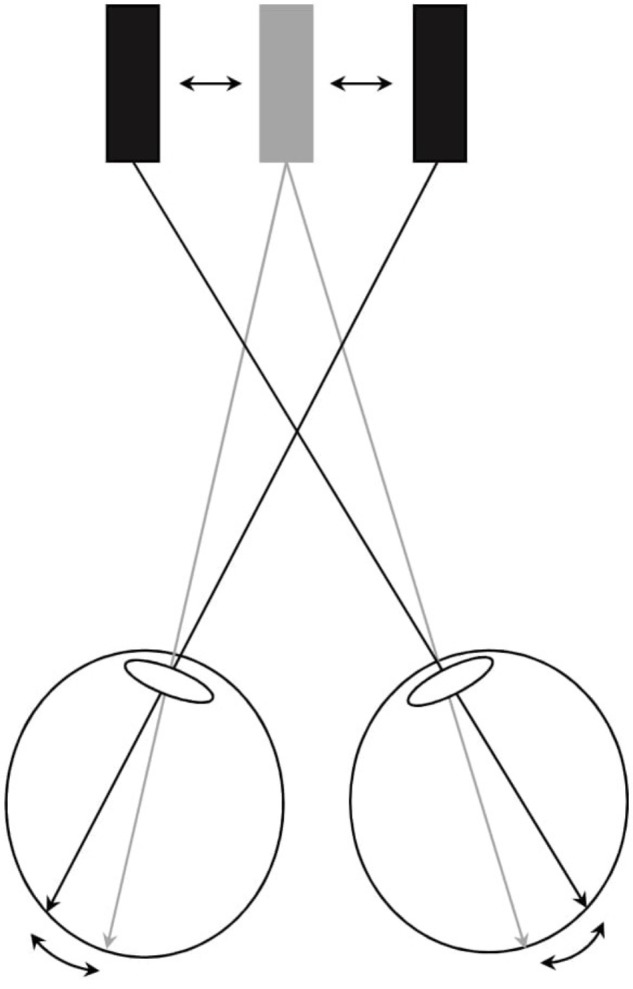
Visualization of stimulus to convergence during conflicting stimulus-to-accommodation and stimulus-to-vergence eye-movements. Participants fixated upon the bar presented separately to the left and right eyes through polarized filters. A computer setup was used that enabled horizontal vergence load to be induced dynamically. When convergence eye movements were stimulated, a conflicting stimulation to accommodation and vergence was induced since accommodation needs to be maintained at 1.54 diopters (i.e., the distance to the bar stimuli projected on the screen, illustrated by the grey stimulus bar) to see the bar clearly, and not to the corresponding distance of the vergence angle of the eyes (illustrated by the black bars). By increasing the horizontal distance between the two images (black bars), convergence was stimulated and by reducing the horizontal distance, divergence was stimulated. If subjects did not comply with the vergence stimulus change by producing compensatory divergence/vergence eye movements, then diplopia resulted.

**FIGURE 2:**
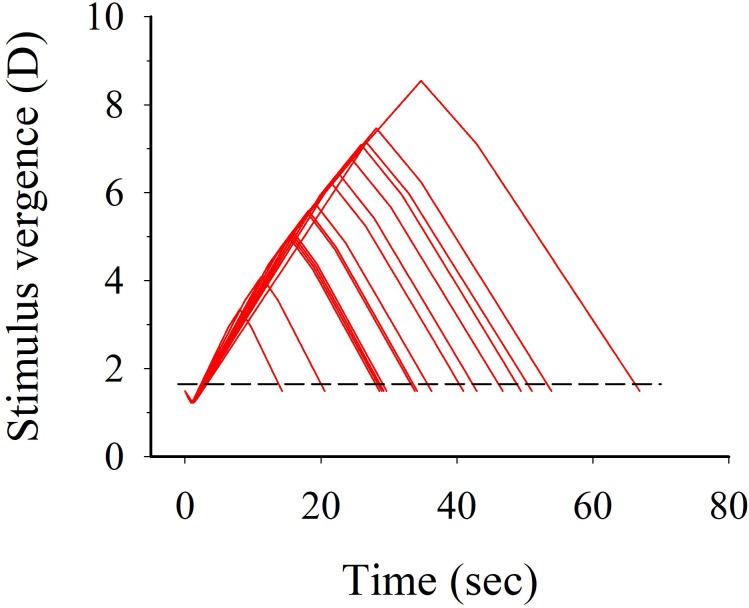
Plots of stimuli (D) to convergence during conflicting stimulus-to-accommodation and stimulus-to-vergence eye-movements presented to the subjects during the two 10-min visual conditions *Conv* and *Cc*. The individual magnitude of stimulation to convergence eye-movements shown herein constitute 80% of the mean threshold value for diplopia (convergence break point). Figure only shows one cycle of horizontal oscillations in added convergence load relative to the starting stimulus distance 1.65 diopters (stippled reference line).

The vision task consisted of sustained foveal fixation on a horizontal bar stimulus (40 mm × 8 mm) generated by a stationary computer (ASUS Eee Box PC Model nr: EB1501P) running a Linux Gnome (version 2.30) operating system. The horizontal bar stimuli were displayed on a 3D screen (Fujitsu P23T-6 FPR 3D). Participants fixated upon the bar, which was presented separately to the left and right eyes through polarized filters. The computer setup enabled the stimulus to convergence/divergence eye-movements to fluctuate in a sinusoidal fashion while allowing the stimulus to accommodation to remain unchanged. By using polarized glasses, fusion was possible, and a 3D image of the vertical bar stimuli appeared. By separating the bars, and increasing their horizontal distance (i.e., the bar to the left on the screen seen by the right eye, and the bar to the right of the screen seen by the left eye), convergence eye-movements were stimulated, and by reducing the horizontal distance of the bars, divergence eye-movements were stimulated. When vergence eye movements were stimulated, a conflicting stimulation to accommodation, and stimulation to vergence, was induced, since accommodation needed to be maintained at 1.54 D (i.e., the distance to the screen) to see the bar clearly, and not to the corresponding distance of the vergence angle of the eyes. When there was no conflict present between accommodation and convergence, the convergence demands corresponded to 9.77 PD (i.e., 4.88 PD to each eye; refer to **Figure 1**). In *Conv* and *Cc* conditions, the magnitude of stimulus to convergence/divergence eye-movements, which was presented to the participants, varied individually (**Figure 2**). Prior to the experiment, the individual threshold for the fusion breakpoint (i.e., the perception of diplopia) was established using a staircase method in which the magnitude of stimulation to convergence during conflicting accommodation/convergence eye-movements was increased incrementally until diplopia occurred. Ten separate diplopia thresholds (convergence break points) were obtained in this manner and an individual mean was calculated from these measurements. The individual magnitude of stimulation to convergence eye-movements used in the experiment constituted 80% of this mean threshold value. To test the hypothesis that the individual ability to execute relatively larger amplitudes convergence eye-movements during conflicting stimulus-to-accommodation and stimulus-to-vergence eye-movements impacted upon dlPFC oxygenation (hypothesis 2), a variable *Convergence load_Low/High_* was created which dichotomized the participant’s diplopia thresholds (convergence breakpoints) into two subcategories: (1) those exhibiting an individual threshold below the group median (“*Low convergence load”*) and (2) those above the group median (“*High convergence load”*). After the experiment, ten additional individual post-test diplopia thresholds were recorded to obtain an estimate of potential oculomotor learning effects. In two out of the four conditions, *Ctrl* and *Cog*, participants were presented with no disparity and consequently, a single fused bar stimulus was consistently perceived.

### Functional Near-Infrared Spectroscopy (fNIRS)

Non-invasive measurements of regional hemodynamic changes over the dlPFC were quantified with a near-infrared spectrometer (fNIRS: PortaLite mini, Artinis Medical Systems, Zetten, Netherlands). Mean penetration depth was ∼12.5 mm into the cortex and the sampling frequency was 10 Hz. An increase in brain activity is generally assumed to reflect an increase in local HbO_2_ and decreases in HHb as based on a mechanism known as neurovascular coupling (Ferrari and Quaresima, 2012; Scholkmann et al., 2014). A decrease in HbO_2_ response may also reflect neuronal suppression, or alternatively the re-distribution of blood flow (Ferrari and Quaresima, 2012). Neuronal activation is thought to be accompanied by the expansion (vasodilatation) of perfused blood vessels and by an increased portion of perfused vessels (recruitment) (Obrig et al., 2010). tHb is related to cerebral blood volume (HbO_2_ minus HHb).

The experiment was carried out in a in a dimmed laboratory room with controlled levels of air temperature and noise. The fNIRS probe was placed on the PFC on the right dlPFC. A modified version of the 10–20 system (Oostenveld and Praamstra, 2001) was used for placement of the probe. In order to suppress surrounding light, the probe was covered with a piece of black cloth. The dedicated software application Oxysoft (Artinis Medical Systems, Zetten, Netherlands) was used to collect, store, and visualize the data. The Oxysoft software was used to delineate the baseline and the experimental periods from one another. The Oxysoft software was also used for computing relative measures of HbO_2_, HHb, and tHb values, as based on the modified Lambert Beer law (Scholkmann et al., 2014). Real time calculation and visualization of oxy-, deoxy-, and total hemoglobin allowed validation of the data acquisition process. The Oxysoft software permits export of raw data in unformatted text format. This Oxysoft function was used to make the data available in MATLAB (MATLAB R2017a, MathWorks, Natick, MA, United States) where it was visualized in time series plots and further inspected for artifacts. Data processing was completed with a custom-written MATLAB script.

Several steps were taken in the computation of baseline-subtracted local HbO_2_, HHb and tHb. First, prior to performance in the vision task, a baseline was recorded for 2-min during which participants were instructed to sit quiet in solitude and rest with their eyes closed. Then, mean values were calculated from -25 to -5 s immediately preceding each experimental condition to create baseline values, which were then subtracted from all HbO_2_, HHb, and tHb values measured during the corresponding visual task, thus providing ΔHbO_2_, ΔHHb, and ΔtHb. Finally, the individual means for ΔHbO_2_, ΔHHb, and ΔtHb were determined across each of the 10 s periods, focusing on 0, 2, 4, 6, 8, and 10 min points of the visual task time. Averaging of fNIRS raw data, in the 10 s windows, was done in order to cancel out systemic contamination influences (heart rate and breathing waves). Equipment failure led to the loss of data in one case. The final data set consisted of *Ctrl* (*n* = 19), *Conv* (*n* = 19), *Cog* (*n* = 19), and *Cc* (*n* = 19).

### Experience of Mental Fatigue

Before the experiment began, and after each experimental condition, the participants were asked to rate “*To what extent do you feel mentally strained*?” on the Nasa Task Load index scale varying from “*very little*” (=0) to “*Very much*” (=10). The individual ratings of mental fatigue immediately after participation in the experimental conditions were used to characterize how mentally demanding, and tiring, the experiment was.

### Statistical Testing

Statistical analyses were performed using IBM SPSS 24.0 for Windows (SPSS Inc., Chicago, IL, United States) and the significance level was set at α = 0.05. All variables were first tested for normality using the Kolmogorov–Smirnov test. The choice of subsequent statistical tests was based on the distribution of data, as detailed below.

#### Linear Mixed Model Analysis

Linear mixed effects model (LMM) analyses were used to investigate whether the level of dlPFC hemodynamic activity, reflected by changes in ΔHbO_2_, ΔHHb, and ΔtHb, differed significantly over time during added convergence load, and conflicting accommodation/vergence stimuli, with and without added cognitive load. We chose LMM analysis because the data was organized as a two-level hierarchical structure with repeated measurements within the first level of data (experimental condition). Multi-level analysis by LMM was used to control for contextual dependencies within the data. The hierarchical structure featured ‘experimental condition’ at level 1 and ‘subjects’ at level 2. Time was a nested variable within subjects. Multi-level modeling was carried out with fixed (linearly independent) effects and random intercepts. Time was modeled as a repeated measurement using an autoregressive model (AR[1]) process. LMM analysis provides a general framework for the analysis of data arising from a hierarchical time series and also relaxes other key assumptions associated with traditional regression models (Fitzmaurice et al., 2004). The current LMM is equivalent to a linear model in which the intercepts are allowed to vary across experimental conditions (Field, 2015). A component was added to the intercept that measures the variability in intercepts, *u_0j_*. Therefore, the intercept changes from *b_0_* to *b_0_* + *u_0j_*. This term estimates the intercept of the overall model fitted to the data, *b_0_*, and the variability of intercepts around that overall model, *u_0j_*. The overall model becomes:

$$Y_{ij}=(b_{0}+u_{0j})+b_{1j}X_{ij}...+\varepsilon_{ij}$$

The *j*s in the equation reflect levels of the variable over which the intercepts varies, in this case the experimental conditions. *𝜀* denotes the error term. Signal contamination, from, e.g., optode movements, and other potential sources to measurement error increases the error term which in turn decreases the ratio of explained variance to unexplained variance. It is not possible to compute effect size values from a LMM analysis. As an alternative information criterion, the so called -*2 Restricted Log Likelihood*, smaller the better, is used to compare the fitness level of alternative models (Field, 2015).

The dependent variables in the mixed level analysis model were changes in ΔHbO_2_, ΔHHb, and ΔtHb. The independent variables were *Time* (0, 2, 4, 6, 8, and 10 min) which were coded as a categorical variable with minute 0 as a reference value, *Condition* (*Ctrl, Conv, Cog*, and *Cc*) coded as a categorical variable with *Ctrl* as a reference value and *Convergence load_Low/High_* coded as a categorical variable with *Convergence load_Low_* as a reference value. The LMM analysis models simultaneously tested the effect of *Time, Condition, Convergence load, Time* × *Condition, Time* ×*Convergence load, Condition* ×*Convergence load*, and *Time* × *Condition* ×*Convergence load* using one model each for changes in ΔHbO_2_, ΔHHb, and ΔtHb. Univariate fixed effects and pairwise comparisons, based on estimated marginal means (i.e., linear predicted values), were used to test various aspects of hypotheses 1–3. Adjustments for multiple comparisons were performed with the Bonferroni method. The following statistical hypotheses were considered:

(1) The effect of *Time* (0, 2, 4, 6, 8, and 10 min) on ΔHbO_2_, ΔHHb, and ΔtHb should be significant when pooled over the four conditions (*Ctrl, Conv, Cog*, and *Cc*) and *Convergence load_Low/High_*.
(2) The effect of Condition (*Ctrl, Conv, Cog*, and *Cc*) on ΔHbO_2_, ΔHHb, and ΔtHb should be significant when pooled over *Time* and *Convergence load_Low/High_*. Testing of this hypothesis involved partial testing of hypothesis 1. If external exposure influenced the amount of ΔHbO_2_, ΔHHb, and ΔtHb in *Conv*, *Cog*, or *Cc*, then hemodynamic and/or blood volume changes should be different when compared to *Ctrl*.
(3) The effect of *Time* ×*Condition* should be significant when pooled over *Convergence load_Low/High_*. Testing of this hypothesis involved testing of hypothesis 1. If external exposure influenced the level of change for ΔHbO_2_, ΔHHb, and ΔtHb in *Conv*, *Cog*, or *Cc*, then the extent of changes at 0, 2, 4, 6, 8, or 10 min should be different when compared to *Ctrl*.
(4) The effect of *Convergence load_Low/High_* on changes in ΔHbO_2_, ΔHHb, and ΔtHb should be significant when pooled over *Time* and *Condition*. Testing of this hypothesis involved partial testing of hypothesis 2. Notably, this test included pooled data from condition *Ctrl* and *Cog*, in which no convergence load was present. The variable *Convergence load_Low/High_* may, however, contain additional effects other than oculomotor load, such as high or low level of sustained attention.
(5) The effect of *Time* × *Convergence load* (low or high) on changes in ΔHbO_2_, ΔHHb, and ΔtHb should be significant when pooled over the different conditions. Testing of this hypothesis involved partial testing of hypothesis 2. If internal exposure impacted on the amount of ΔHbO_2_, ΔHHb, and ΔtHb in the sub-category of high convergence load then changes should be different at 0, 2, 4, 6, 8, or 10 min when compared to the sub-category of low convergence load. Notably, this test also included pooled data from condition *Ctrl* and *Cog*, in which no convergence load was present.
(6) The effect of *Condition* × *Convergence load_Low/High_* on changes in ΔHbO_2_, ΔHHb, and ΔtHb involved testing of hypothesis 2. If internal exposure of *Convergence load_Low/High_* impacted upon hemodynamic activity and/or blood volume changes when pooled over time, then changes should be different between low and high convergence load in condition *Conv* and *Cc* only. This is because conditions *Ctrl* and *Cog* did not contain any stimulus to convergence load. Testing of this hypothesis thus complements the earlier analytic step by considering convergence load as a variable that specifically exerts an oculomotor effect.
(7) The effect of *Time* × *Condition* × *Convergence load* on changes in ΔHbO_2_, ΔHHb, and ΔtHb involved testing of hypothesis 3. If time related ΔHbO_2_, ΔHHb, and ΔtHb changes were influenced by a combination of added convergence demands and added cognitive demands, as compared to conditions with only added convergence demands, only added cognitive load, or no added load, then the amount of hemodynamic and/or blood volume changes at a given time, across different conditions, should differ within the low or high convergence load sub-categories.

## Results

### Vergence Range Values Pre- and Post- experimental Performance

The group mean (*n* = 20) stimulus dioptric value for the convergence break point prior to the experiment was 7.71 D (range: 3.86–11.02, SD: 1.93). Within the *Convergence load_Low_* sub-group this mean was 6.10 D (*n* = 10, range: 3.86–7.12, SD; 1.06) and for the *Convergence load_High_* subgroup, it was 9.27 D (*n* = 10, range: 7.72–11.02, SD: 1.17). The correlation coefficient, *r*_xy_, between individual stimulus dioptric values associated with the convergence break point prior to the experiment *versus* after the experiment was *r*_xy_ 0.83 (*p* < 0.0001, slope = 1.13, *r*^2^ = 0.68). When the correlation coefficient was recalculated within the low/high convergence stimulus subgroups it reached significance only within the *Convergence load_High_* subgroup (*load_High_*: *r*_xy_: 0.71, *p* < 0.05, *load_Low_*: *r*_xy_: 0.19, *p* > 0.05). The overall difference between pre-test and post-test dioptric stimulus values was 0.58 D (*n* = 20, *SD*: 1.50, range: -1.92–2.62). For the *Convergence load_Low_* subgroup, the difference was 0.07 D (range: -1.92–2.25). For the *Convergence load_High_* subgroup, the difference was 1.11 D (range: -1.03–2.62). The pre-post difference was significant only for the *Convergence load_High_* subgroup (Wilcoxon Signed Rank test, *T* = 48, *p* < 0.05).

### *n*-Back Test Performance

The median reaction time was 1.51 s in *Cog* (range: 0.90–1.68 s, *n* = 19) and 1.55 s (range: 1.17–2.40 s, *n* = 19) in *Cc*. The median proportion of correctly recalled items was 90% in *Cog* (range: 24–97%) and 85% in *Cc* (range: 6–100%). The median number of missed items was 3 (range: 1–25) in *Cog* and 5 (range: 0–31) in *Cc*; refer to **Table 1**. Non-parametric Wilcoxon Signed Rank tests showed no differences in RT across conditions *Cog* and *Cc* (*z* = 1.39, *p* > 0.05), a significant difference in a higher number of correct responses in *Cog* relative to *Cc* (*U* = 30, *z* = -1.97, *p* < 0.05) and a lower number of missed items in *Cog* relative to *Cc* (*U* = 106, *z* = 1.97, *p* < 0.05). When the differences in *n*-back performance between conditions were tested separately within the low/high convergence demand subgroups, we identified significant differences within the *Convergence_Low_* subgroup (*n* = 9) for the number of correct responses, which was higher in *Cog* relative to *Cc* (*z* = -1.963, *p* < 0.05), and for the number of missed responses, which was lower in *Cog* relative to *Cc* (*z* = 1.963, *p* < 0.05). In the *Convergence_High_* subgroup (*n* = 10), these aspects of *n*-back test performance were not significantly different from each other (*p* > 0.05).

**Table 1:** Median RT (min–max values), the correct number of responses (min–max) values and the number of misses (min–max) in the entire study population (Total), and in the *Convergence_Low_* (*n* = 9) group and the *Convergence_High_* (*n* = 10) group.

Condition	Total (*n* = 19)			*Convergence_Low_* (*n* = 9)			*Convergence_High_* (*n* = 10)		
	RT	Correct	Misses	RT	Correct	Misses	RT	Correct	Misses
*Cog*	1.51	30	3	1.48	23	10	1.54	30	3
	(0.90–1.68)	(8–32)	(1–25)	(0.90–1.67)	(8–32)	(1–25)	(0.93–1.68)	(24–32)	(1–9)
*Cc*	1.55	28	5	1.51	23	10	1.59	30	3
	(1.17–2.40)	(2–33)	(0–31)	(1.17–1.76)	(2–33)	(0–31)	(1.30–2.40)	(21–33)	(1–12)

*Cog, n-back test performance only; Cc, convergence load during conflicting accommodation/vergence eye-movements to avoid diplopia concurrent with n-back test performance.*

### Mental Fatigue

After performing in the *Ctrl*, *Conv, Cog*, and *Cc* conditions, mental fatigue averaged 2.90 (STD: 2.72, range: 0.1–9.90, *n* = 10), 3.44 (*SD*: 2.30, range: 0.4–8.10, *n* = 10), 3.32 (*SD*: 2.46, range: 0.30–9.30, *n* = 10) and 4.45 (*SD*: 2.82, range: 0.70–8.90, *n* = 10), respectively. The low convergence load subgroup exhibited more mental fatigue immediately after the experiment in condition *Ctrl* relative to the high convergence load subgroup [*t* = 3.17(18), *p* = 0.004]. In the other three experimental conditions, mental fatigue ratings immediately after the experiment did not differ between the low and high convergence load subgroups (*p* > 0.10).

### dlPFC Oxygenation

**FIGURE 3:**
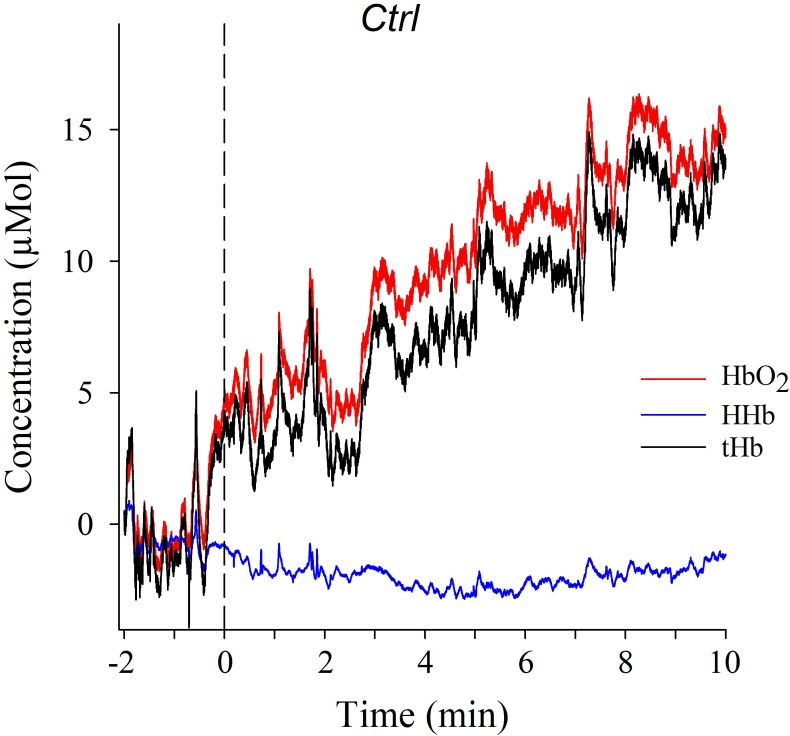
Typical NIRS time series plot of individual changes in HbO_2_, HHb, and tHb during the 2-min baseline condition (to the left of the stippled vertical line), and during the 10-min visual *Ctrl* condition (to the right of the stippled vertical line).

**Figure 3** shows a representative example of fNIRS data from one of the participants in condition *Ctrl*. The individual traces indicated good signal quality in that systemic pulse oscillations and respiratory waves are clearly visible in all recordings.

#### Overall Comparisons Across Viewing Conditions and Temporal Patterns of dlPFC Oxygenation

**FIGURE 4:**
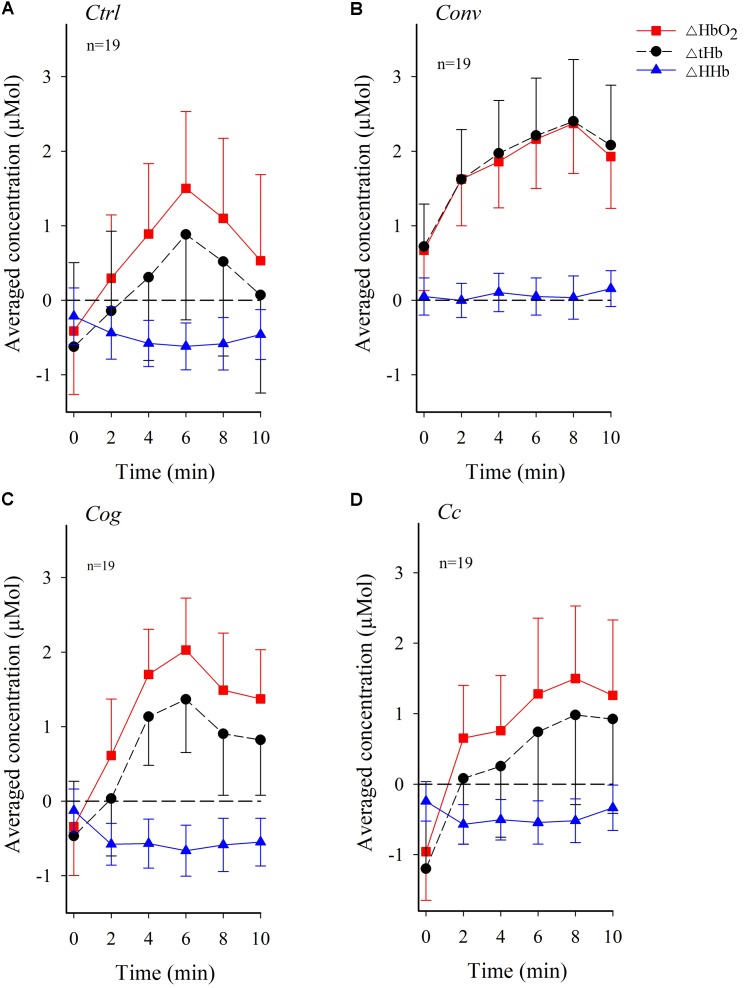
Temporal pattern of changes in the concentration of oxygenated hemoglobin (ΔHbO_2_) and deoxygenated hemoglobin (ΔHHb) along with variations in total hemoglobin (ΔtHb) in dlPFC across visual conditions (figure shows mean values, and standard errors). **(A)** Control condition *Ctrl*. **(B)** Convergence load condition *Conv*. **(C)** Cognitive control condition *Cog*. **(D)** Combined Convergence and Cognitive load, condition *Cc*. The stippled horizontal reference line denotes no change in baseline subtracted hemodynamic activity.

**Figure 4** shows group means of activity across the four conditions over time. A systematic increase in ΔHbO_2_ and ΔtHb activity immediately at the onset of the experiment continuing through minute 6 in *Ctrl* and *Cog*, and minute 8 in *Conv* and *Cc*, can be observed in **Figure 4**. After the peak in activity, a decelerating trend can be observed, particularly in conditions *Ctrl* and *Cog*.

#### Low and High Convergence Load and Temporal Patterns of dlPFC Oxygenation

**FIGURE 5:**
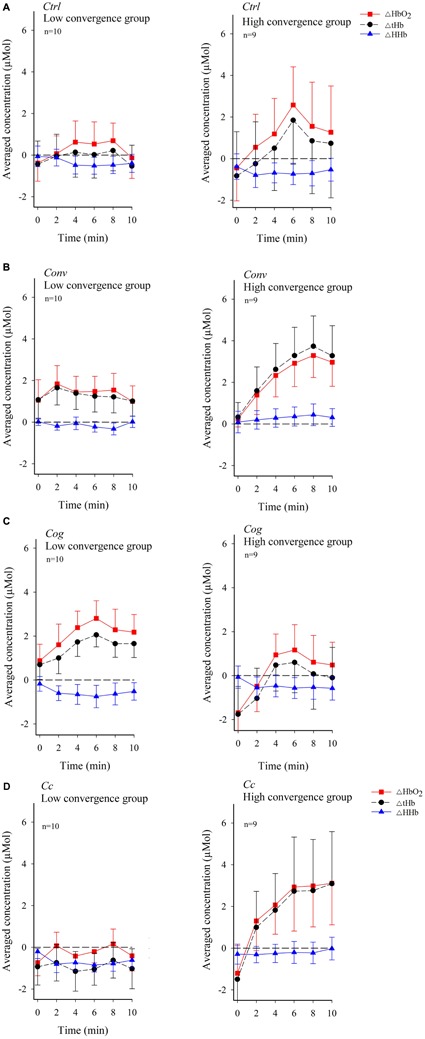
Temporal pattern of changes in the concentration of oxygenated hemoglobin (ΔHbO_2_) and deoxygenated hemoglobin (ΔHHb) along with variations in total hemoglobin (ΔtHb) in dlPFC across visual conditions for each participant in the low and high convergence load subgroup (figure shows mean values and standard errors). **(A)** Control condition *Ctrl*. **(B)** Convergence load condition *Conv*. **(C)** Cognitive control condition *Cog*. **(D)** Combined convergence and cognitive load, condition *Cc*. The stippled horizontal reference line denotes no change in baseline subtracted changes.

**Figure 5** shows temporal patterns of dlPFC oxygenation in the low or high convergence load subgroups across experimental conditions. A systematic increase in ΔHbO_2_ and ΔtHb immediately at the onset of the experiment continuing through minute 8 in *Conv*, and minute 6 in *Ctrl*, *Cog*, and *Cc*, can be observed among participants belonging to the high convergence demand subgroup. Participants exposed to low convergence demands in all conditions except *Cog* exhibited a more flattened trend for ΔHbO_2_ and ΔtHb, particularly in condition *Cc*. ΔHHb generally exhibited negative values.

#### LMM Results

There was a fixed effect of *Time* on ΔHbO_2_, ΔHHb, and ΔtHb (*p* < 0.0001 in all cases). Pairwise comparisons, based on estimated marginal means, showed that ΔHbO_2_ values were higher in magnitude for minutes 2–10 relative to minute 0 (*p* < 0.0001), larger in magnitude for minutes 6–8 relative to minute 2 (*p* < 0.001–0.05), larger in magnitude for minute 6 relative to minute 4 (*p* < 0.05). Minute 8 did not differ from minute 6, and minute 10 did not differ from minute 8 (*p* > 0.05 in all cases). Pairwise comparisons, based on estimated marginal means, also showed that ΔHHb values were lower in magnitude for minute 2 (*p* < 0.0001), minute 4 (*p* < 0.05) and minute 6 (*p* < 0.05) relative to minute 0. All other comparisons were not significant (*p* > 0.05). Furthermore, pairwise comparisons showed that ΔtHb values were larger in magnitude for minutes 2–10 relative to minute 0 (*p* < 0.0001–0.01 in all cases), larger in magnitude for minute 6 relative to minute 4 (*p* < 0.005). Finally, minute 8 did not differ from minute 6, and minute 10 did not differ from minute 8 (*p* > 0.05 in all cases). A fixed effect of *Time* ×*Convergence load_Low/High_* was apparent for ΔHbO_2_ and ΔtHb (*p* < 0.01 in all cases) but not for ΔHHb (*p* > 0.05). There was no fixed effect of *Time* ×*Condition* ×*Convergence load* on ΔHbO_2_, ΔHHb, or ΔtHb (*p* > 0.05 in all cases). Pairwise comparisons, based on estimated marginal means, showed, however, that ΔHbO_2_ and ΔtHb were lower for the *Convergence_Low_* subgroup when compared to the *Convergence_High_* subgroup during minute 10 in condition *Cc* only (*p* < 0.05) with a trend during minute 6 and 8 (*p* < 0.10). All other *p*’s > 0.10. See **Table 2**.

**Table 2:** Tests of univariate fixed effects.

Dependent variable		HbO_2_^∗^			HHb^∗∗^			tHb^∗∗∗^		
Source	Numerator df	Denominator df	*F*	Sig.	Denominator df	*F*	Sig.	Denominator df	*F*	Sig.
Intercept	1	17.60	4.64	0.04	17.33	2.24	0.15	17.38	1.19	0.29
Time	5	326.81	11.65	<0.001	330.62	5.13	<0.001	327.32	7.02	<0.001
Condition	3	56.64	0.640	0.59	57.96	1.66	0.186	55.92	1.22	0.31
Convergence load	1	17.60	0.23	0.64	17.33	0.13	0.722	17.38	0.23	0.64
Time × Condition	15	326.81	0.88	0.59	330.62	0.72	0.769	327.32	0.73	0.756
Time × Convergence load	5	326.81	3.35	0.006	330.62	0.94	0.458	327.32	3.74	0.003
Condition × Convergence load	3	56.64	1.79	0.16	57.96	0.63	0.598	55.92	1.71	0.18
Time × Condition × Convergence load	15	326.81	0.67	0.81	330.62	1.21	0.265	327.32	0.80	0.68

*^∗^Information criteria, smaller the better, -2 Restricted Log Likelihood = 1717. ^∗∗^ = 801. ^∗∗∗^1777.*

## Discussion

The results from this study indicate that hemodynamic activity recorded over dlPFC with fNIRS change over time, are impacted by convergence load, and do not differ between experimental conditions. These three main findings, discussed more in detail below, indicate that fNIRS can be used to assay the degree to which the accommodation/vergence system is strained during visually demanding near work.

### dlPFC Oxygenation Changes Over *Time*

All three fNIRS parameters were influenced by time. The concentration of ΔHbO_2_ and ΔtHb increased in magnitude over time while the concentration of ΔHHb decreased in magnitude immediately after minute 0 and then remained stable below 0 throughout minutes 2–10. The leveling off observed for mean ΔHbO_2_ and ΔtHb may be related to a change in mental effort and/or or a change in goal setting (Hockey, 2011). These significant effects of *Time* set the stage for a more specific investigation of research hypotheses 2–3 outlined in the following sections.

### Effect of *Convergence Load* on dlPFC Oxygenation

A comparatively low and static dlPFC oxygenation in condition *Cc* was associated with a smaller, and over time, also unchanging capacity to execute convergence eye-movements under conflicting stimulus-to-accommodation and stimulus-to-vergence eye movements. Small fusional reserves, idiosyncratic cross-coupling of accommodation and convergence, and/or a pre-existing condition of mental fatigue, may have rendered these subjects unable and/or unwilling to execute larger amplitudes of convergence eye-movements. For these reasons, the relatively low and static dlPFC oxygenation in this subgroup could be related to a low level of mental effort. In this scenario, the relative absence of hemodynamic activity was probably caused by a lack of tonic dlPFC neuronal discharge over time as related to the absence of active supervisory oculomotor processes. A conscientious effort to overcome the persistent difficulties to execute convergence eye-movements would have probably activated the cortical machinery in the dlPFC and raised the metabolic needs of the cortical neurons. The behavioral differences characterizing the low convergence load subgroup, the perturbed *n*-back test performance, and the lower convergence break thresholds, could alternatively be the consequence of an insufficient recruitment of working memory related brain circuits (Granet et al., 2005; Ehlis et al., 2008).

Over time, larger individual convergence break point values were associated with a systematic increase in ΔHbO_2_ and ΔtHb when pooled over the four experimental conditions. The changes in ΔHHb concentration were not significant. The increased concentrations of ΔHbO_2_ presumably reflect an increase in local arteriolar vasodilatation, which increases local cerebral blood flow and cerebral blood volume (Ferrari and Quaresima, 2012). The concomitant lack of change in ΔHHb follows a pattern seen in previous studies (Ferrari and Quaresima, 2012). As conflicting stimulus to accommodation/vergence eye-movements were only present in conditions *Conv* and *Cc*, this outcome suggests the existence of a global dlPFC effect of “energization” (Cieslik et al., 2015) or an extended view of working memory as a monitoring process of action in the face of sensory incongruence (Fink et al., 1999). More specifically, prior to the fNIRS recording, those individuals with more vigilance may have ended up with larger diplopia thresholds because they happened to have more mental resources available at the time of the testing and *vice versa*. The tendency to be “vigilant” or mentally “fatigued” may have prevailed during performance in the four experimental conditions and to some extent impacted upon dlPFC oxygenation in conditions which also lacked a convergence load stimulus (Duncan, 2001).

A systematic increase in ΔHbO_2_ and ΔtHb over time, when tested separately within each of the four experimental conditions, occurred only within condition *Cc* and among those subjects which were exposed to a larger stimulus to conflicting accommodation/vergence eye-movements during the very end of the 10-min period. The same line of reasoning as that used to explain the lack of between-condition effects (see below, “Lack of Differences in dlPFC Oxygenation Between *Conditions*”) may explain why the effect of convergence load only surfaced in the *Cc* condition. In the *Conv* condition, subjects were not shielded from boredom and the consequences of this probably obscured the effect of convergence load among the two subcategories of low or high convergence load (Duncan, 2001). In particular, the low convergence load subcategory of participants, which exhibited more mental fatigue following their performance in the *Ctrl* condition and performed slightly worse in the *n*-back test in *Cc* compared to *Cog*, may have been more prone to allocate PFC resources in order to stay concentrated on the task at hand (Duncan, 2001).

### Lack of Differences in dlPFC Oxygenation Between Conditions

Results were not impacted by *Condition* or *Time* ×*Condition*, in other words, changes in ΔHbO_2_ and ΔHHb and ΔtHb did not differ between conditions regardless of whether concurrent *n*-back performance was performed or not, or if subjects were exposed to concurrent convergence load. The reason for similarity in the temporal patterns of changes in ΔHbO_2_ and ΔHHb and ΔtHb may reside in similar task-related attention demands which inadvertently led to PFC activation. Inherent in both experimental conditions lay identical requirements to “sit down stationary,” “fixate upon the screen” and “be quiet.” Hence, the effect of *Time* in *Ctrl*, and perhaps also in *Conv*, presumably was caused by compatible tonic dlPFC neuronal discharge as related to the active maintenance of task-related attention templates in working memory. For example, in the face of boredom, mentally under-stimulated and/or fatigued subjects may have been forced to launch cortical resources related to the inhibition of undesired behavioral responses (Hockey, 1997; Faber et al., 2012; Nakagawa et al., 2013; Tanaka et al., 2014; Wang et al., 2016). If this premise is true, this evoked activity may have obscured our between- condition comparisons. More specifically, the *Ctrl* condition would not have been as neutral as we intended. In this scenario, dlPFC neurons systematically increased their tonic discharge over time to counteract mental fatigue and/or boredom. The *n*-back test probably recruited dlPFC and served as a shield against mental boredom/fatigue in the *Cog* and *Cc* conditions. In *Ctrl* and *Conv*, this shield was not available. This circumstance probably forced the subjects to allocate PFC resources to stay concentrated on the task so that they could be task compliant. Task demands in *Ctrl* and *Conv*, for this reason, may have been compatible with those in *Cog* and *Cc*. Collectively, these circumstances probably acted to obscure the expected between-condition effects in activation between conditions *Ctrl* and *Conv, and between Cog* and *Cc*.

#### Lack of Task Interference Effects on dlPFC Oxygenation During Concurrent Convergence and Mental Load

No significant changes in the concentrations of ΔHbO_2_, ΔHHb, or ΔtHb were observed among the low/high convergence loads in condition *Cc* when compared to condition *Conv*. According to the adaptive coding model of PFC function, this could have occurred because prefrontal functions are notoriously difficult to compare and contrast across different experimental tasks because the population of cortical neurons adjust their function to match the requirements of any given task undertaken (see section “*n*-Back Test Performance in *Cog* and *Cc* Conditions”; Duncan, 2001).

### Convergence Eye-Movements During Conflicting Stimulus-to-Accommodation and Stimulus-to-Vergence Eye Movements

The task to respond to the horizontal oscillations in stimulus to convergence eye-movements during conflicting stimulus-to-accommodation, and stimulus-to-vergence eye movements, showed satisfactory reproducibility as evidenced by both pre- and post-test comparisons. Individual horizontal convergence fusional reserves (break points) during normal viewing conditions vary from person to person, target type, and method used, and generally fall within the range of 2–15 D when measured at near range (Scheiman et al., 2003). This range also encapsulates the current convergence near point values which averaged 7.71 D (or 12.5 PD). Post-test measurements of convergence near point values suggested a small but significant increase in ability to execute fusional vergence eye-movements in the high, but not low, convergence load subgroup. This may be indicative of short term plasticity and learning in the accommodation/vergence system (see section “Short Term Visual Plasticity in the dlPFC in a Network for Oculomotor Control”).

### *n*-Back Test Performance in *Cog* and *Cc* Conditions

The great majority of the subjects performed the *n*-back test in a satisfactory manner, in other words, with less than 10% error; this indicates a high degree of task compliant performance. A tendency was found for the low convergence load group to perform slightly worse in condition *Cc* relative to condition *Cog*. As condition *Cc* entailed concurrent convergence load, dual task interference seems a likely explanation for this result (Leone et al., 2017). Dual task interference occurs when the simultaneous performance of two different tasks results in the deterioration of performance in one or both tasks. Since the low convergence load group reported more mental fatigue following performance in condition *Ctrl*, this subgroup of participants may have been more vulnerable to dual task interference than the high convergence load subgroup.

### The Moderating Effect of Mental Fatigue on dlPFC Oxygenation

The lack of difference in fatigue ratings between the low and high convergence load subgroups following performance in the Cc condition makes compensatory effort per se an unlikely cause behind the increased concentration of ΔHbO_2_ and the concomitant increase in ΔtHb, which were both observed to take place over time in only the high convergence load subgroup. The individual convergence stimulus amplitudes, determined before the test, were likely tailored to the level of mental fatigue experienced by the participants. Hence, both the high and low convergence load subgroups scaled their convergence effort to be compatible with one another. Only in the absence of convergence load did the low convergence stimulus group manifest with more mental fatigue than the high convergence load.

### Short Term Visual Plasticity in the dlPFC in a Network for Oculomotor Control

Diplopia thresholds increased in magnitude only in the *Convergence load_High_* subcategory of participants (as evident in the pre- and post-task comparisons). Analogous to the findings presented by Ramsay et al. (2014), the increased dlPFC oxygenation over time may be caused by short-term activity-dependent plasticity and learning in a network for oculomotor control subservient of convergence/divergence coordination eye-movements of which the dlPFC plays a part. In this scenario, the dlPFC increased its metabolic demands over time because of a systematic increase in tonic discharge of cortical neurons, the increased recruitment of cortical cells, and an increase in their firing rate. Chan et al. (2004) reported greater gray matter volume in strabismic adults relative to normal controls in areas consistent with the PFC and attributed these changes to plasticity in the oculomotor regions to compensate for the cortical deficits in the visual processing areas. The conflicting stimulus-to-accommodation, and stimulus-to-vergence, introduced by the horizontal oscillations in retinal disparity could trigger the first step of such cortical plasticity by perturbing the normal synergy between accommodation/vergence. Deficient visual near work in this scenario has the effect of recruiting supervisory oculomotor control processes to aid the perception action cycle and to uphold its integrity. These “back up” procedures are supervised by the dlPFC and other brain centers outside of the field of view (e.g., Gamlin and Yoon, 2000). The increased oxygenation in the dlPFC may be a manifestation of such processes.

### Methodological Considerations and Limitations

In future studies, it would be desirable to design experimental conditions so that they are equal in all aspects except for the oculomotor provocation of interest. This practice will optimize statistical analyses between condition comparisons. The classification and separation of fNIRS signals, moreover, represents a challenging task (Scholkmann et al., 2014). The signal measured by fNIRS, as well as other brain imaging methodologies (e.g., fMRI and PET), are contaminated with signal components not associated with functional brain activity per see (Scholkmann et al., 2014). Low- pass filtering of the fNIRS raw data in the current study, at a frequency of 0.08–0.1 Hz, should therefore have been practiced, as this is expected to remove the non-evoked systemic components in the fNIRS signal (e.g., respiration and pulse-related physiological noises) (Scholkmann et al., 2014). Motion artifacts on fNIRS signals should, for the same reason, be removed by dedicated software. The use of only one probe in the current study is associated with additional limitations. Mislocalization of the dlPFC area is a possibility. Mislocalization problems may have impacted on the statistical power and obscured, e.g., the between conditions comparisons. An increased risk for a Type II error (declaring a difference “not statistically significant” even when there really is a difference) is a possibility. Without omnibus correction, a few significant effects arose on ΔHbO_2_ and ΔtHb, which actually suggested differences between conditions. Pairwise comparisons based on estimated marginal means showed that ΔtHb was higher for Convergence load High in condition *Cc* when compared to condition *Cog* during minute 6. Pairwise comparisons based on estimated marginal means also showed that ΔHbO_2_ was lower for Convergence load Low in condition *Cc* when compared to condition *Cog* during minute 6. There is an inherent tradeoff between protecting against Type I errors (declaring differences “statistically significant” when they are in fact just due to a coincidence of random sampling) and Type II errors. The use of only one probe in the current study, moreover, precluded hemispheric lateralization of dlPFC oxygenation during convergence efforts, virtually unknown at present, to be addressed. A widened study protocol including probes over the visual cortex would also have been desirable. Barceló et al. (2000) provided anatomical, electrophysiological and behavioral evidence that dlPFC regulate neuronal activity in extrastriate visual cortex (BA 18/19) during visual discrimination. dlPFC seems to be critically involved in holding a representation of the sought-after stimulus (e.g., a fused visual target as opposed to an unfused diplopic target) in working memory in order to guide behavior (e.g., convergence) (Barceló et al., 2000). A comparison of the evoked visual cortex hemodynamic activity between individuals with high or low convergence ability could have helped to elucidate the underlying mechanisms.

## Future Research

A pertinent long-term research aim of our visual neuroergonomic research agenda is to deliver novel information relating to the extent to which both young and old brains work to meet contemporary visual physical/mental work demands. To accomplish this, more dedicated pre-processing of fNIRS signal and more channels are required to better capture the significant hemodynamic events occurring in the PFC, not only in the left or right dlPFC, but also in other areas, e.g., in the visual cortex. A larger range of age is also desirable in the future studies.

### Conclusion

HbO_2_ and tHb, recorded over the dlPFC with fNIRS, offer a unique method with which to assay the degree to which the visual system is strained over time during demanding near work conditions involving accommodation/convergence effort. Model predictions based on fNIRS data recorded over PFC are expected to result in a deeper understanding of the phenomenon of visual fatigue. More knowledge of the underlying mechanisms will be useful in preventing and/or ameliorating the symptoms subjects who are afflicted by binocular and accommodative anomalies during demanding near work suffer from.

## Author Contributions

HR, MF, CZ, and GE contributed substantially to the conception and design of the work and to the acquisition, analysis, and interpretation of data for the work. HR, MF, GE, RB, JM, and CZ contributed to the initial draft of this manuscript in terms of intellectual content and approved the final version. All authors agreed to be accountable for all aspects of the work in ensuring that questions related to the accuracy or integrity of any part of the work were appropriately investigated and resolved.

## Conflict of Interest Statement

The authors declare that the research was conducted in the absence of any commercial or financial relationships that could be construed as a potential conflict of interest.

## Abbreviations

ΔHbO_2_: baseline subtracted oxygenated hemoglobin
ΔHHb: baseline subtracted deoxygenated hemoglobin
ΔtHb: Baseline subtracted total hemoglobin volume
*Cc*: added convergence load during conflicting accommodation/vergence eye movements concurrent with *n*-back test performance
*Cog*: *n*-back test performance
*Conv*: added convergence load during conflicting accommodation/vergence eye movements
*Ctrl*: a control condition involving no convergence or cognitive load
dlPFC: right dorsolateral pre-frontal cortex (BA 46)
HbO_2_: oxygenated hemoglobin
HHb: deoxygenated hemoglobin
NIRS: near infrared spectroscopy
rCBV: regional cerebral blood volume
tHb: total hemoglobin volume
