# Corrigendum: Prefrontal Cortex Oxygenation Evoked by Convergence Load Under Conflicting Stimulus-to-Accommodation and Stimulus-to-Vergence Eye-Movements Measured by NIRS

**DOI:** 10.3389/fnhum.2018.00384

**Published:** 2018-09-19

**Authors:** Hans O. Richter, M. Forsman, G. H. Elcadi, R. Brautaset, John E. Marsh, C. Zetterberg

**Affiliations:** ^1^Department of Occupational and Public Health Sciences, Faculty of Health and Occupational Studies, Centre for Musculoskeletal Research, University of Gävle, Gävle, Sweden; ^2^Institute of Environmental Medicine, Karolinska Institutet, Stockholm, Sweden; ^3^Department of Health and Caring Sciences, Faculty of Health and Occupational Studies, University of Gävle, Gävle, Sweden; ^4^School of Optometry, Karolinska Institutet, Stockholm, Sweden; ^5^Environmental Psychology, Department of Building, Energy, and Environmental Engineering, University of Gävle, Gävle, Sweden; ^6^School of Psychology, University of Central Lancashire, Preston, United Kingdom; ^7^Section of Occupational and Environmental Medicine, Department of Medical Sciences, Uppsala University, Uppsala, Sweden

**Keywords:** attention fatigue, accommodation, compensatory effort, convergence, disparity, near infrared spectroscopy (NIRS), time series analysis, visual ergonomics

In the original article, there was a mistake in Figure 5A (left panel) as published.

**Figure 5:**
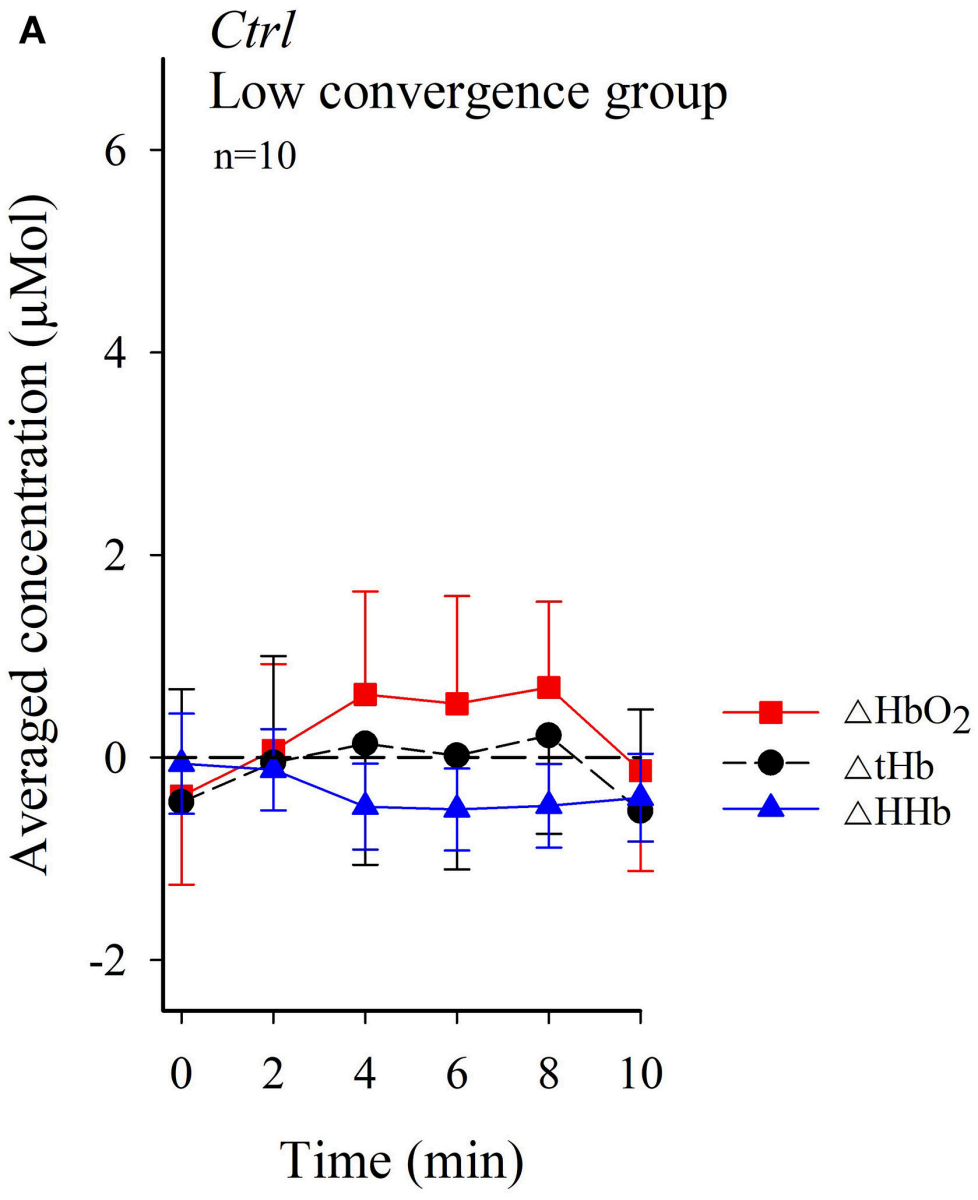


Figure 5A (left panel) should show Ctrl Low convergence group, not Conv Low convergence group. The corrected Figure 5A (left panel) appears below. The authors apologize for this error and state that this does not change the scientific conclusions of the article in any way.

The original article has been updated.

## Conflict of interest statement

The authors declare that the research was conducted in the absence of any commercial or financial relationships that could be construed as a potential conflict of interest.

