# Supplementary material for: Prefrontal Cortex Oxygenation Evoked by Convergence Load Under Conflicting Stimulus-to-Accommodation and Stimulus-to-Vergence Eye-Movements Measured by NIRS
Source: Front Hum Neurosci. 2018 Jul 30;12:298. doi: 10.3389/fnhum.2018.00298 (PMC6077206; doi:10.3389/fnhum.2018.00298)
Supplement: Supplementary file 1 [file Table_1.DOCX]

| **Nr.** | **Gender** | **Age (years)** | **Unaided VA** | | | **Refraction - sphere** | | **Cylinder** | | **Axis** | | **BVA** | | | **Phoria 6 m** | | **Phoria 40 cm** | | **NPC (D)** | **NPA (D)** | | | **Colour Vision** |
| --- | --- | --- | --- | --- | --- | --- | --- | --- | --- | --- | --- | --- | --- | --- | --- | --- | --- | --- | --- | --- | --- | --- | --- |
|  |  |  | **OD** | **OS** | **Bino** | **OD** | **OS** | **OD** | **OS** | **OD** | **OS** | **OD** | **OS** | **Bino** |  |  |  |  |  | **OD** | **OS** | **Bino** | **Ishihara** |
| 1. | W | 28 | 1.2 | 1 | 1.2 | 0.25 | 0.25 | -0.25 | -0.25 | 5 | 175 | 1.2 | 1.2 | 1.5 | 2.0 | eso | 0.7 | eso | ToN | 11 | 12 | 13 | N |
| 2. | W | 20 | 1.2 | 1.2 | 1.2 | 0.75 | 0.75 | 0.25 | 0.25 | 115 | 70 | 1.2 | 1.2 | 1.2 | 10.2 | exo | 12.5 | exo | ToN | 11 | 10 | 12 | N |
| 3. | W | 29 | 1.5 | 1.2 | 1.5 | 0 | 0.25 | 0 | 0 | 0 | 0 | 1.2 | 1.2 | 1.5 | 0.9 | exo | 7.0 | exo | ToN | 12 | 12 | 14 | N |
| 4. | W | 19 | 1.2 | 1.2 | 1.2 | 0.25 | 0.25 | 0.25 | 0.25 | 170 | 180 | 1.2 | 1.5 | 1.5 | 2.3 | eso | 3.1 | exo | ToN | 14 | 14 | 14 | N |
| 5. | W | 18 | 1.2 | 1.2 | 1.2 | 0.25 | 0.25 | -0.25 | -0.25 | 130 | 65 | 1.2 | 1.2 | 1.5 | 1.5 | exo | 8.5 | exo | ToN | 13 | 14 | 14 | N |
| 6. | M | 23 | 0.9 | 0.8 | 0.9 | -0.5 | -0.25 | -0.5 | -0.5 | 5 | 35 | 1.0 | 1.0 | 1.0 | 2.8 | exo | 9.0 | exo | ToN | 13 | 13 | 16 | N |
| 7. | M | 32 | 1.2 | 1.5 | 1.5 | 0.75 | 0.75 | -0.5 | -0.25 | 100 | 100 | 1.5 | 1.5 | 1.5 | 4.2 | exo | 5.6 | exo | 7 | 9 | 10 | 10 | N |
| 8. ^a^ | W | 22 | - | - | - | - | - | - | - | - | - | - | - | - | - | - | - | - | - | - | - | - | - |
| 9. | M | 34 | 0.9 | 1 | 1 | 1.25 | 1.25 | -0.75 | -0.75 | 105 | 95 | 1.2 | 1.2 | 1.5 | 0.7 | exo | 8.9 | exo | 5 | 8 | 8 | 9 | N |
| 10. | W | 43 | < 0.05 | < 0.05 | < 0.05 | -4.0 | -4.0 | -1.0 | -0.5 | 150 | 25 | 1.0 | 1.0 | 1.0 | 0.5 | exo | 8.5 | exo | ToN | 6 | 6 | 8 | N |
| 11. | W | 18 | 1.2 | 1.5 | 1.5 | 0.25 | 0.25 | 0 | 0 | 0 |  | 1.2 | 1.2 | 1.5 | 1.0 | eso | 0.7 | exo | ToN | 11 | 10 | 12 | N |
| 12. | W | 44 | 0.6 | 0.8 | 1.0 | 1.75 | 1.75 | -1.0 | -0.75 | 85 | 130 | 1.2 | 1.2 | 1.5 | 6.4 | exo | 4.2 | exo | 6 | 6 | 5 | 5 | N |
| 13. | W | 32 | 1 | 1.2 | 1.2 | 0 | 0 | 0 | 0 | 0 | 0 | 1.2 | 1.2 | 1.2 | 3.5 | eso | 4.3 | exo | ToN | 12 | 12 | 13 | N |
| 14. | W | 36 | 1.5 | 1.5 | 1.5 | 0.25 | 0.75 | 0 | -0.5 | 0 | 170 | 1.5 | 1.5 | 1.5 | orto |  | 9.5 | exo | 5 | 8 | 8 | 10 | N |
| 15. ^a^ | W | 24 | - | - | - | - | - | - | - | - | - | - | - | - | - | - | - | - | - | - | - | - | - |
| 16. | W | 24 | 1.2 | 0.5 | 0.7 | -1.0 | -0.75 | 0 | -0.25 | 0 | 75 | 1.2 | 1.2 | -1.5 | orto |  | 3.0 | exo | ToN | 13 | 13 | 13 | N |
| 17. ^a^ | W | 31 | - | - | - | - | - | - | - | - | - | - | - | - | - | - | - | - | - | - | - | - | - |
| 18. ^a^ | W | 21 | - | - | - | - | - | - | - | - | - | - | - | - | - | - | - | - | - | - | - | - | - |
| 19. ^a^ | M | 28 | - | - | - | - | - | - | - | - | - | - | - | - | - | - | - | - | - | - | - | - | - |
| 20. | W | 39 | 0.3 | 0.1 | 0.2 | -1.8 | -1.5 | -0.25 | -0.5 | 60 | 145 | 1.2 | 1.2 | 1.2 | 3.8 | eso | 5.9 | eso | ToN | 5 | 5 | 6 | N |

**Table 1.** Description of participants and results from optometric evaluation (n =15).

^a^ = not available. Unaided VA = Unaided visual acuity. BVA = Best corrected visual acuity. orto = Ortophoria. Eso = Esophoria. Exo = Exophoria. NPC = Near point of convergence. ToN= tip of noise. N = Normal. † = Average of ten separate diplopia thresholds (convergence break points) using the horizontal bar stimuli displayed on the 3D screen (see section 2.4. Vergence stimulation, for details).
